# Portable Aptasensor Based on Parallel Rolling Circle Amplification for Tumor‐Derived Exosomes Liquid Biopsy

**DOI:** 10.1002/advs.202403371

**Published:** 2024-06-26

**Authors:** Yaqin He, Xianghu Zeng, Ying Xiong, Congcong Shen, Ke Huang, Piaopiao Chen

**Affiliations:** ^1^ Department of Laboratory Medicine Med+X Center for Manufacturing National Clinical Research Center for Geriatrics West China Hospital Sichuan University Chengdu Sichuan 610041 China; ^2^ College of Chemistry and Material Science Sichuan Normal University Chengdu Sichuan 610068 China

**Keywords:** aptamer, dual targeting, exosome, parallel RCA, portable homogeneous detection

## Abstract

Here, a separation‐free and label‐free portable aptasensor is developed for rapid and sensitive analysis of tumor‐derived exosomes (TEXs). It integrated a parallel rolling circle amplification (RCA) reaction, selective binding of metal ions or small molecules to nucleic acid‐specific conformations, and a low‐cost, highly sensitive handheld fluorometer. Lung cancer, for example, is targeted with two typical biomarkers (mucin 1 and programmed cell death ligand 1 (PD‐L1)) on its exosomes. The affinity of aptamers to the targets modulated the amount of RCA products (T‐Hg^2+^‐T and cytosine (C)‐rich single‐stranded DNA), which in turn affected the fluorescence intensity of quantum dots (QDs) and methylene blue (MB). The results revealed that the limit of detection (LOD) of the handheld fluorometer for cell‐derived exosomes can be as low as 30 particles mL^−1^. Moreover, its specificity, sensitivity, and area under the curve (AUC) are 93% (14/15), 92% (23/25), and 0.956, as determined by the analysis of 40 clinical samples. Retesting 16 of these samples with the handheld fluorometer yielded strong concordance between the fluorometer results and those acquired from clinical computed tomography (CT) and pathology.

## Graphical abstract

A portable tumor‐derived exosomes aptasensor is constructed based on the parallel rolling circle amplification (RCA) reaction and dual fluorescence indicators by targeting mucin 1 and programmed cell death ligand 1 (PD‐L1) on the surface of tumor exosomes.

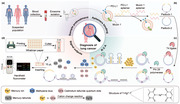

## 1 Introduction

According to the International Agency for Research on Cancer (IARC), there were ≈2.2 million additional cases of lung cancer worldwide in 2020, ranking second in the incidence of malignant tumors.^[^ ^1^ ^]^ Given that the 5‐year survival rate of early lung cancer can exceed 70%, it is critical to achieve early diagnosis.^[^ ^2^ ^]^ Compared to tissue biopsy, which is associated with certain operational requirements and risks, liquid biopsy can non‐invasively detect the levels of cancer biomarkers such as tumor‐derived exosomes (TEXs), circulating tumor DNA and cells in body fluids.^[^ ^3^, ^4^, ^5^, ^6^, ^7^, ^8^ ^]^ This positions it as a more useful tool for the early diagnosis and surveillance of cancer. Compared to the other two, TEXs have garnered extensive attention owing to their high abundance and stability in the blood.^[^ ^9^, ^10^ ^]^ At present, immunogold and protein blotting assays are considered the clinical standard methods for identifying specific proteins on TEXs but have not yet been able to yield quantitative results and are time‐consuming and laborious.^[^ ^11^ ^]^ Thus, there is an urgent need to pioneer easy‐to‐use and sensitive quantitative assays for exosomes.

In recent research, emerging sensors based on electrochemistry, thermophoresis, microfluidic chips, surface‐enhanced Raman spectroscopy, and fluorescence have been used for quantitative analysis of TEXs.^[^ ^12^, ^13^, ^14^, ^15^, ^16^ ^]^ Among them, fluorescence stands out owing to its simple experimental facilities, non‐invasive nature, and easy miniaturization. Currently, its homogeneous detection largely relies on the labeling of fluorescent moieties, and it remains challenging to develop a label‐free and separation‐free homogeneous method. Indeed, it requires the collaboration of specific recognition probes, efficient nucleic acid amplification, and effective fluorescence signal amplification. With outstanding portability and stability and high affinity to the target, nucleic acid aptamers outperform antibodies and peptides under homogeneous conditions.^[^ ^17^, ^18^, ^19^ ^]^ Nucleic acid amplification techniques such as catalyzed hairpin assembly, hybridization chain reaction, rolling circle amplification (RCA), *etc*. are employed to further enhance detection sensitivity.^[^ ^20^, ^21^, ^22^, ^23^, ^24^ ^]^ Among them, RCA is highly regarded for its efficiency, programmability and specificity. Considering the proteins expressed on exosomes are not specific, dual‐targeted combined assays are more useful for accurate cancer diagnosis.^[^ ^25^, ^26^ ^]^ The selection of parallel RCAs that can trigger the amplification of both strands at the same time not only makes the template design simpler, but also achieves multiple uses of one enzyme, thus optimizing enzyme utilization and reaction efficiency. Finally, metal ions or molecular beacons are paired with specific binding to nucleic acid‐specific conformations to achieve signal output and amplification. This encompasses the embedding of metal ions in mismatched base pairs and the interaction of MB with cytosine (C)‐rich single‐stranded DNA (ssDNA).^[^ ^27^, ^28^ ^]^ This synergistic interaction of these components paves the way for the development of labeling‐free and separation‐free homogeneous methods.

With the assistance of homogeneous system, portable detection is more accessible. Commonly used point‐of‐care testing (POCT) methods, including glucometers, blood pressure meters, weight scales, and smartphone software all require the reconversion of signals such as distance, weight, and color to digital signals. Inspired by this, a low‐cost, high‐endurance handheld fluorometer was pioneered to directly determine fluorescence signals. With a compact structure comparable to the size of a cell phone, light‐emitting diode (LED) monochromatic light as the excitation light source. A long lifetime, low power consumption, and stable luminescence, silicon light‐emitting diode serving as detectors can respond to light signals to achieve rapid and sensitive readings. This study aimed to integrate it with an exosome‐based homogeneous aptamer sensor.

**Scheme 1:**
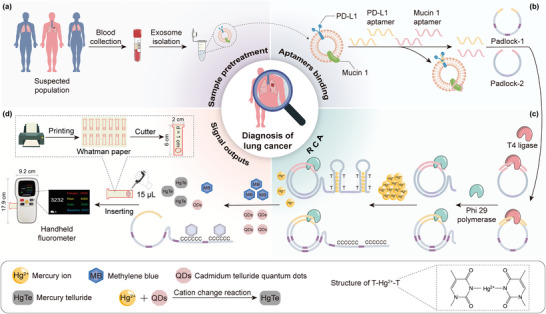
Schematic diagram for separation and detection of exosomes. a) Separation of exosomes from peripheral blood samples of clinical lung cancer patients. b–d) Exosome‐triggered parallel RCA homogeneous assay and portable analysis.

Herein, aptamers, parallel RCA reaction, and fluorescent molecular signal amplification were combined to design label‐free and separation‐free sensitive, portable TEX aptasensor (**Scheme** **1**). Taking lung cancer as an example, two typical biomarkers (mucin 1 and programmed cell death ligand 1 (PD‐L1)) on TEXs were selected as targets. Not only due to they are overexpressed, but they have an important role in tumor progression, metastasis, and immune evasion.^[^ ^29^ ^]^ The core of the sensor was that the high affinity of the aptamer to the protein influenced subsequent RCA reactions and regulated RCA products. The dual‐target, simultaneous quantification strategy allowed for accurate measurement of TEX concentrations. Coupled with the specific binding of quantum dots (QDs) and methylene blue (MB) to T‐Hg^2+^‐T and C‐rich ssDNA, two fluorescent signals were generated, respectively. This was further combined with the handheld fluorometer for resource‐limited environments. The results revealed that a minimum of 30 particles mL^−1^ of TEXs could be detected by both instruments. Next, 40 and 16 cases clinical samples were analyzed by the fluorometer and handheld fluorometer, respectively. The results all showed strong concordance with clinical images, highlighting the clinical utility of the aptasensor.

## 2 Results and Discussion

### 2.1 Analytical Principle

The objective of this study was to simultaneously quantitatively assess two exosomal membrane proteins (mucin 1 and PD‐L1) via dual fluorescent signals (QDs and MB) (Scheme 1). Mucin 1 and PD‐L1 have both been established to be overexpressed in lung cancer TEXs, and their levels correlate with lung cancer development and progression. Therefore, the combined detection can aid in the precise diagnosis of cancer.^[^ ^11^, ^30^, ^31^ ^]^ Exosomes were initially isolated from tumor cell supernatants or blood samples using conventional ultracentrifugation (Scheme 1a). Then, the dual‐target detection system was constructed based on two aptamers as recognition probes, an extension of T‐Hg^2+^‐T hairpin structure and C‐rich ssDNA through parallel RCA reactions as a bridge, and the simultaneous use of QDs and MB (Figure S1, Supporting Information) as fluorescence amplification signals.

Parallel RCA triggered the amplification of both strands simultaneously, which not only allowed for efficient use of the enzyme, but also a simple sequence design that allowed for dual‐target detection with only four sequences. Specifically, the assay system relied on the extremely high affinity between the aptamer and mucin 1 and PD‐L1 (dissociation constants *K_d_* = 0.135 nm and 4.7 nm, respectively).^[^ ^32^, ^33^ ^]^ This dissociated the binding of the aptamer to the padlocked probe and prevented the subsequent RCA reaction from occurring (Scheme 1b). Two 5′‐phosphorylated padlock probes were first designed according to the aptamers, and the complementary sequences were thoroughly encoded to acquire the corresponding products (Table S1, Supporting Information). The padlock probes consisted of two modular sequences: a complementary sequence linking the 5′ and 3′ termini to the aptamer, and a long strand containing either A or G bases and some spacer sequences. In this way, T‐T DNA or C‐C DNA tandem repeat sequences were amplified. In order to enhance the interaction of Hg^2+^ and MB to the bases, six consecutive thymine (T) or C bases fragments were required for each segment. In the absence of exosomes, the padlock probes undergo an RCA reaction assisted by T4 DNA ligase and phi29 DNA polymerase to extend a large amount of ssDNA rich in T and C (Scheme 1c). Among them, the T‐T DNA can protect the fluorescence of QDs by selectively trapping Hg^2+^ to form stable T‐Hg^2+^‐T complex. Meanwhile, the presence of mucin 1‐positive exosomes inhibited the RCA reaction, thereby limiting the production of T‐Hg^2+^‐T. Free Hg^2+^ underwent cation‐exchange reaction (CER) with QDs, quenching the latter. A similar strategy was used for PD‐L1 detection. In the absence of exosomes, the extended C‐rich ssDNA hindered the electronic transition of the MB and affected its luminescence. In contrast, MB fluorescence was restored in the presence of PD‐L1‐positive exosomes. Eventually, QD signals were decreased, whereas MB signals were increased in positive samples.

The fluorescence results obtained could be detected not only by a fluorimeter but also by the self‐developed handheld fluorometer (Scheme 1d). Of note, it was comparable to the size of a cell phone and could be used with non‐fluorescent paper cut into the corresponding shape (Schemes S1 and S2, Supporting Information). The instrument consisted of a housing, a control system, and a display. A deep ultraviolet 280 nm LED lamp was utilized as a monochromatic excitation device, using a band‐pass filter, whilst a silicon photodiode served as a fluorescence detector. Whatman chromatography paper was chosen as the test strip considering the materials loading capacity and storage conditions. Rapid fluorescence detection was achieved by dropping 15 µL of solution onto the test strip circle. Moreover, the sensitivity of the instrument had been improved by shortening the optical path, increasing the light transmission when the test strip was moistened, and using the high‐power light source, etc. (Scheme S3, Supporting Information). Overall, the handheld fluorometer expands the choice of exosomal POCT.

### 2.2 Feasibility of Exosome Analysis Method

**Figure 1:**
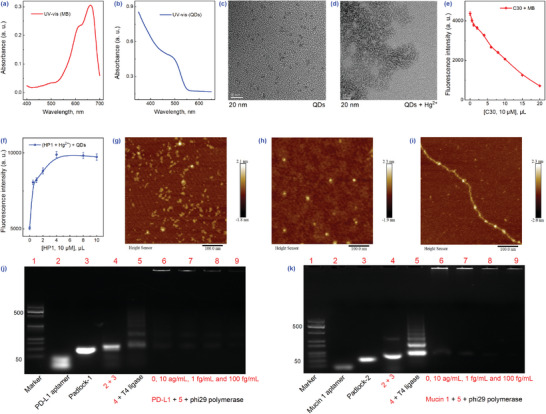
Characterization and feasibility of detection methods. UV absorption spectra of MB a) and QDs b). TEM of QDs c), and QDs + Hg^2+^ d), scale bar: 20 nm. e) Fluorescence signal response of MB at different C30 concentrations. f) Fluorescence signal response of QDs when generating T‐ Hg^2+^‐T using different HP1 concentrations. Representative AFM images of aptamers and padlock probes prior to ligation g), following ligation h), and after RCA reaction i) (PD‐L1 as an example, scale bar: 100 nm). Agarose gel electropherograms of RCA reactions between PD‐L1 j) and mucin 1 k), respectively. The error bars were estimated from triplicate measurements.

The UV‐vis spectroscopy of MB and QDs was first examined. As illustrated in **Figure** **1**a, MB displayed two characteristic absorption peaks at 662 nm (monomer form) and 605 nm (dimer form).^[^ ^34^ ^]^ In contrast, the absorption peaks of QDs were at ≈475 nm (Figure 1b). The concentration of QDs stock solution used in this study was estimated to be 12 µm.^[^ ^35^ ^]^ Transmission electron microscopy (TEM) images depicted the shift in QDs from homogeneous dispersion to distinct agglomeration after reacting with Hg^2+^ (Figure 1c,d), which implied that the CER was triggered. Earlier studies have reported that C‐rich ssDNA (C30) interacted with MB in enthalpy‐driven processes.^[^ ^28^ ^]^ Thus, as the concentration of C30 increased, the signaling of MB was significantly attenuated (4000 reduced to 700, Figure 1e). On the other hand, the signal of QDs increased with an increase in HP1 chains, reaching the maximum signal at 4 µL HP1 chains (Figure 1f). Thus, the specific recognition of T‐Hg^2+^‐T and Hg^2+^ by QDs was validated. The aforementioned two selective reactions were the basis for subsequent detection.

Following this, an atomic force microscope (AFM) and gel electrophoresis were employed to demonstrate the feasibility of the RCA reaction. Taking PD‐L1 as an example, the aptamer and padlock probe showed evenly distributed dots prior to ligation (Figure 1g). As delineated in Figure 1h, circular shapes appeared following linkage, signaling the formation of circular templates. Notably, extended DNA nanowires were clearly visible after the introduction of polymerase and dNTPs solution (Figure 1i), signifying the successful generation of ultra‐long ssDNA. The phenomenon was similarly corroborated using gel electrophoresis. In other words, Figure 1j portrayed migrating bands slowly appearing in lane 5, with extra bands forming at the top in lane 6, suggesting the generation of circular templates and the successful reaction of RCA, respectively. Importantly, from lane 6 to lane 9, the color of the bands progressively faded as the concentration of PD‐L1 increased (same goes for mucin 1, Figure 1k). The aforestated results conjointly confirmed the feasibility of adding proteins to inhibit the RCA reaction.

### 2.3 Analytical Performance of Mucin 1 and PD‐L1

**Figure 2:**
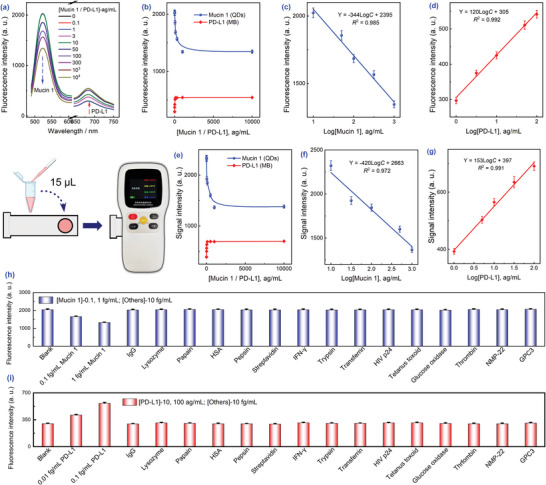
Analytical performance of dual‐signal simultaneous detection of mucin 1 and PD‐L1. Fluorescence spectrum a) and values b) at different protein concentrations. Linear fits for different mucin 1 c) and PD‐L1 d) concentrations. Fluorescence values e) and corresponding linear fits f,g) were similarly obtained by handheld instrumental assays. h,i) Selectivity assessment. Error bars were obtained based on three replicate experiments.

In order to determine the feasibility of the method, several experimental conditions were optimized to yield a satisfactory analytical performance (Figures S2–S4, Supporting Information). According to the experimental principle (Scheme 1), the fluorescence signal of QDs decreased with increasing concentrations of mucin 1, whilst that of MB increased with increasing concentrations of PD‐L1 (**Figure** **2**a). As anticipated, the fluorescence values were linearly related to the logarithm of protein concentration (Log *C*). A linear range of 10 −1000 ag mL^−1^ and 1–100 ag mL^−1^ were obtained for mucin 1 and PD‐L1, respectively (Figure 2b–d). For use outside the laboratory, the portability of the handheld instrument was excellent. To further determine its detection ability, 15 µL the reaction solution was placed inside the circle of the test strip, which was immediately inserted into the instrument for reading. The results also yielded a good linear relationship (Figure 2e–g). The limit of detections (LODs) were estimated from the signal‐to‐noise ratio (*S/N*, n = 3) as 3 ag mL^−1^ (mucin 1) and 0.5 ag mL^−1^ (PD‐L1), respectively, which are comparable to fluorometry (the LODs were 4 and 0.3 ag mL^−1^ for the two proteins, respectively).^[^ ^36^, ^37^ ^]^ At the same time, specificity was assessed by detecting the abundance of proteins including transferrin, human serum albumin (HSA), immunoglobulin G (IgG), *etc*.) and cancer targets (including interferon‐gamma (IFN‐*γ*), glypican 3 (GPC 3), *etc*.) in plasma. The signals of the potential interferents at 10 fg mL^−1^ were comparable to the blank group (Figure 2h,i). Conversely, low concentrations of the target proteins (100 ag mL^−1^ mucin 1 or 10 ag mL^−1^ PD‐L1) significantly altered fluorescence signals. Taken together, our findings demonstrated that the high affinity of the aptamer to the protein conferred good specificity to the method, which was essential for subsequent clinical exosome detection.

Of note, our method eliminated the need for tagging and separation compared to non‐homogeneous detection methods. Compared with homogeneous methods, the method possessed excellent sensitivity, owing to the combined effect of nucleic acid amplification, nanomaterial cascade amplification, and electron transfer from MB. Moreover, antibody capture and complex material synthesis were not required. Hence, the dual‐target synchronized detection strategy had significant advantages over existing homogeneous protein assays (Table S2, Supporting Information).^[^ ^25^, ^38^, ^39^, ^40^ ^]^

### 2.4 Analytical Capability of Exosomes

**Figure 3:**
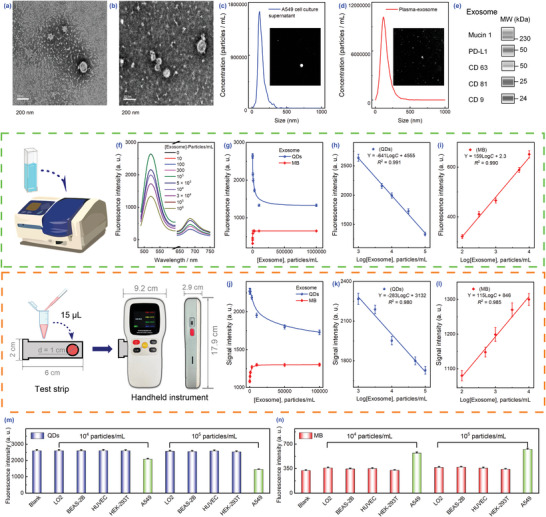
Characterization and detection of isolated exosomes. TEM images and NTA of exosomes derived from A549 cells a,c) and plasma samples b,d). e) Capillary western immunoassay of cell‐derived exosomes. Fluorescence peak patterns f), QDs, and MB signal values g) and their corresponding linear fits h,i) at different exosome concentrations. j–l) Fluorescence values detected by the handheld instrument and the corresponding linear fits. Cell selectivity assessment of QDs m) and MB n) signals. Error bars were obtained based on three replicate experiments.

The upregulation of PD‐L1 and mucin 1 on the surface of lung cancer TEXs enabled the method to be further applied to exosomes extracted from A549 cells. Exosomes were first isolated from cell culture supernatant and blood plasma via ultracentrifugation and subsequently characterized for morphology, particle size, and associated protein expression profiles. TEM images illustrated that both exosomes exhibited a peculiar cup‐shaped morphology with diameters in the range of 50–140 nm (**Figure** **3**a,b). And it was visualized that the concentration of exosomes was higher in plasma. The average concentrations of cell supernatant exosomes and plasma exosomes detected by nanoparticle tracking analysis (NTA) were 6 × 108 exosomes mL^−1^ and 2.8 × 1010 particles mL^−1^, respectively (Figure 3c,d). It was consistent with the TEM results. Capillary western immunoassay detected the expression levels of mucin 1 and PD‐L1, as well as other positive markers (CD9, CD63, CD81) (Figure 3e). The above results indicated the successful isolation and high purity of the target exosomes.

Subsequently, the detection performance of the system was evaluated using cellular exosomes. The results uncovered that both fluorescence signals exhibited good linear correlation with the Log *C*. Importantly, the QDs signals were negatively correlated in the range of 103–105 particles mL^−1^ (*R* ^2^ = 0.991), while MB increased linearly with 102–104 particles mL^−1^ (*R* ^2^ = 0.990) (Figure 3f–i). The linear range of protein concentration was in agreement with that of exosome concentration. Using this method, the detection sensitivity of PD‐L1 was superior to that of mucin 1, and therefore, the corresponding exosome sensitivity was also superior. In addition, LODs were determined to be 200 particles mL^−1^ and 30 particles mL^−1^, respectively, based on the triple *S/N*. Similarly, extending the application of the handheld fluorometer to the cellular level, the detection process was consistent with that of proteins. The linear range ultimately obtained was consistent with that of fluorometry, and the concentration of exosomes could be calculated according to the equation (QDs: Y = −283Log*C* + 3132, MB: Y = 115Log*C* + 846) (Figure 3j–l). In addition, normal cells (human normal hepatocytes LO2 cell, human normal lung epithelial BEAS‐2B cells, Human Umbilical Vein Endothelial Cells (HUVEC), and human embryonic kidney HEK‐293T cells) tested as negative cell lines were found to have fluorescent signal values comparable to those of the control group, indicating satisfactory cell selectivity (Figure 3m,n).

Most of the methods collated in Table S3 (Supporting Information), including microfluidics, electrochemistry, surface‐enhanced Raman spectroscopy (SERS), etc., were non‐homogeneous systems. This work was a homogeneous thermostatic reaction without labeling and separation, which reduced the operation steps and improved the binding efficiency between the target and the aptamer. In addition, more of these methods detected a single biomarker, this work triggered two chain extensions at a time through the delicate parallel RCA design in order to easily realize the detection of dual markers. Moreover, the efficient amplification of RCA combined with the CER of QDs and the electron transfer effect of MB further enhanced the sensitivity.^[^ ^15^, ^16^, ^41^, ^42^, ^43^, ^44^ ^]^ In addition, it can be swiftly and sensitively detected using the handheld fluorometer.

### 2.5 Clinical Practicability of the Exosome Analysis Method

**Figure 4:**
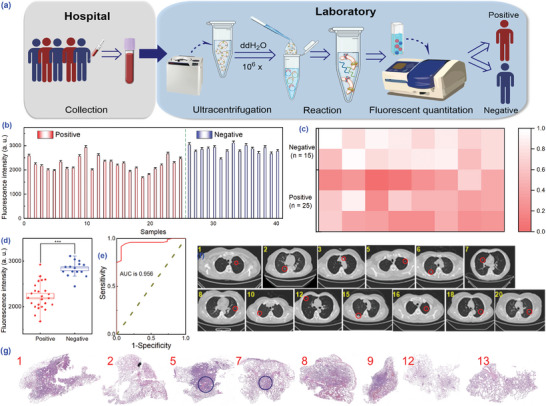
Flowchart and results of the fluorimeter for clinical blood samples. a) Flow of isolation and detection of exosomes in blood samples. b) Fluorescence values of QDs (corresponding to mucin 1). c) Heatmap of normalized QDs signal. Box plot (d, *n* = 25 and 15) and ROC analysis e). Corresponding CT f) and pathologic biopsy findings g) of patients. Error bars were estimated from three repeated measurements. ***p < 0.001 by SPSS 27.0 software through Mann‐Whitney U test.

Clinical blood samples from healthy donors (*n* = 15) and lung cancer patients (*n* = 25) were collected to evaluate the clinical feasibility of the method. After ultracentrifugation for exosome isolation, they were diluted 106‐fold to adjust the concentration within the detection range (**Figure** **4**a). The analytical performance was first evaluated in serum matrix, and it was found that the method still maintained high sensitivity, but the interfering substances in it caused a decrease in the S/N ratio of MB (Figure S5, Supporting Information). However, the MB signal could still aid in differentiation and improve detection rates. Thus, the clinical validation results were a combination of the results from both protein assays (Table S4, Supporting Information). As displayed in Figure 4b,d, the fluorescence signal from positive samples (No. 1–25) was obviously below those of negatives (No. 26–40), suggesting that the former had higher levels of mucin 1 (except for No. 10, 23, and 31). Statistical results *p* < 0.001, indicating statistically significant differences. Besides, a heatmap was constructed to visually distinguish between patients and healthy donors, with the darker colors indicating higher mucin 1 concentrations (Figure 4c). In addition, the receiver operating characteristic curve (ROC) reflected the high accuracy of the method for cancer diagnosis, with an area under the curve (AUC) of 0.956, specificity of 93% (14/15, meant 14 out of 15 negative samples were negative, the value might alter as the sample size expanded), and sensitivity of 92% (23/25, meant 23 out of 25 positive samples were positive) (Figure 4e). Moreover, the exosome diagnostic results were highly consistent with clinical diagnosis (computed tomography (CT) and pathologic biopsy) (Figure 4f,g). For instance, patient No. 1 and No. 5 were both diagnosed as lung cancer, but their CT and pathology tests showed varying degrees of cancer characteristics, resulting in the detection of some differences in their fluorescence values as well. The aforementioned findings strongly validated the utility and reliability of exosome assays in clinical practice.

**Figure 5:**
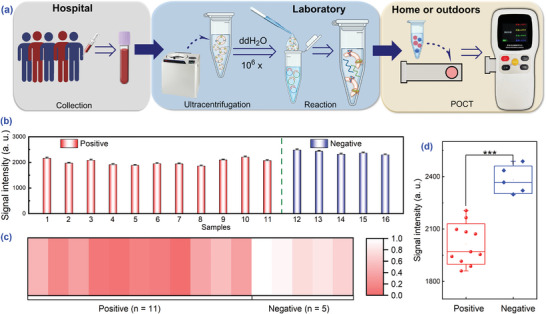
Flowchart and results of the handheld fluorometer for clinical blood samples. a) Flow for sample pre‐processing and POCT. Fluorescence values b), heatmaps c), and box plots d) of QDs. The error bars were obtained from triplicate experiments. ***p < 0.001 by SPSS 27.0 software through Mann‐Whitney U test.

After validating the accuracy of the method, some negative and positive samples in Table S4 (Supporting Information) were selected for further testing using the handheld fluorometer, which is both convenient and accurate, and more suitable for home and outdoor use. Following the termination of the reaction, 15 µL of solution was dropped onto a test strip, which was subsequently inserted into the instrument (**Figure** **5**a). Noteworthily, the handheld fluorometer could not only differentiate between negative and positive samples but also yielded measurements consistent with those from the fluorometer (Figure 5b–d). The clinical results unveiled that the handheld fluorometer only required a dropper, test strips, and a small instrument to complete the detection, making it a potential tool for early disease diagnosis. As a proof‐of‐concept study, it is expected to be promising in clinical practice when combined with commercially available automated reaction and exosome separation instruments.

## 3 Conclusion

In summary, a dual‐target‐triggered parallel RCA homogeneous sensitive aptamer sensor was developed for portable fluorescence quantitative analysis of TEXs. The aptasensor was based on the specific binding of dual aptamers to mucin 1 and PD‐L1, the effective amplification of dual RCA responses, and the selective recognition of specific nucleic acid conformations by dual fluorescence signals (QDs and MB). Finally, it was coupled with a fluorometer and a handheld fluorometer to accommodate various detection scenarios. It is worthwhile emphasizing that both the handheld fluorometer and the fluorometer were capable of detecting cellular exosomes down to 100 particles mL^−1^. The clinical utility of the sensor was initially demonstrated using 40 whole blood samples. Indeed, its specificity, sensitivity, and AUC were 93% (14/15), 92% (23/25), and 0.956, respectively, demonstrating its value for cancer diagnosis. Furthermore, 16 of these samples were randomly selected to validate the accuracy of the handheld fluorometer. The results were in line with those of the fluorometer and clinical diagnosis. The ability to change different aptamers according to the target further demonstrates the versatility of the sensor. However, there was some difference in signal intensity between the two fluorescent molecules in this work, and the pre‐treatment also relied on conventional ultracentrifugation to separate the exosomes. In the future, the use of other signaling molecules or the synthesis of new fluorescent probes will be considered. Meanwhile, automated exosome separation systems such as microfluidics will be combined to further simplify the exosome liquid biopsy process.

## 4 Experimental Section

### Ethics Approval Statement

Clinical whole blood samples were donated and approved by the Biomedical Ethics Committee of West China Hospital of Sichuan University (Chengdu, China, approval number: 20 191 045).

### Materials and Chemicals

All oligonucleotide sequences are shown in Table S1 (Supporting Information) and they were synthesized and purified by Sangon Biotech Co., Ltd (Shanghai, China). Recombinant human mucin 1 and PD‐L1, T4 DNA ligase, deoxynucleotides (dNTPs) mixed solution, phi29 DNA polymerase were obtained by Sangon Biotech Co., Ltd (Shanghai, China). MB was supplied by Thermo Fisher Technology (Waltham, MA, USA). Human lung cancer cells (A549), LO2, BEAS‐2B, HUVEC, and HEK‐293T were provided by Core Facilities of West China Hospital. Phosphate‐buffered saline (PBS) and syringe filters (0.22 µm) were purchased at Gibco Invitrogen Co., Ltd (California, USA). All reagents are at least analytically pure without the need for additional purification steps. Other reagents were available in the Supporting Information.

### Analysis Steps of Exosome

The exosomes from cell supernatants and plasma should be diluted 106‐fold before analysis.

The reaction was performed by adding 10 µm 4 µL Padlock‐1, 10 µm 4 µL PD‐L1 aptamer, and 800 mm 2 µL NaCl to 10 µL of water. Then, the resulting solution was placed in the polymerase chain reaction (PCR) instrument with the settings: 95 °C for 5 min; 65 °C for 2 min; 60 °C for 6 min; 60 to 20 °C, −0.5 °C/30 s; 4 °C for 10 min. Afterward, 40 µL of exosomes were added and reacted for 30 min. For mucin 1, the procedure was identical, except that Padlock‐2 and mucin 1 aptamer were used. Add 40 µL of exosomes and incubate for 30 min.

Thereafter, the two reaction solutions were mixed, followed by the introduction of 10 µL 10 × T4 buffer and 2 µL T4 DNA ligase (5 U µL^−1^), with the reaction proceeding at 37 °C for 1 h. After incubation, T4 ligase was inactivated by heating (65 °C for 10 min). Then, 10 µL 10 × phi29 buffer, 2 µL phi29 DNA polymerase (10 U µL^−1^), 800 mm 10 µL NaCl, 25 mm 4 µL dNTPs, and 20 mg mL^−1^ 1 µL BSA were added, and incubated for 90 min at 37 °C. The mixture was subsequently heated at 65 °C for 10 min. After the adding of 50 µm 5 µL Hg^2+^, the mixture was allowed to react for 1 h to form a T‐Hg^2+^‐T structure. Next, 50 µm 4 µL of MB and 2 µL of QDs stock solution (12 µm) were added and allowed to react for 5 min. Lastly, fluorescence signals were measured.

### Exosome Isolation from Clinical Plasma Samples

Clinical samples were collected in 4 mL of whole blood using disposable closed ethylenediaminetetraacetic acid (EDTA) anticoagulant vacuum blood draws. Immediately within 2 hours, collected whole blood samples were centrifuged at 2.5 × 103 g for 15 min at room temperature to remove platelets, erythrocytes, and leukocytes. Repeat the procedure to obtain platelet‐poor plasma. After that, it was diluted 20‐fold with cold PBS. It was then centrifuged at 104 g for 30 min at 4 °C. For removal of macrovesicles, cellular debris, and other impurities, the liquid was filtered through a syringe‐type membrane (0.22 µm). After filtration, centrifuge at 1.5 × 105 g for 2 h at 4 °C. The resulting precipitate was washed with PBS. This procedure was repeated twice. Finally, the solution obtained was resuspended with PBS and saved at −80 °C.

### Statistical Analysis

Data analysis was obtained by using Origin 2021 and IBM SPSS Statistics 27.0 software. Unless otherwise stated, three replications were performed for each data point on each graph and represented as the mean ± standard deviation (SD). In Figure 4d and Figure 5d, the statistical differences were analyzed using the Mann‐Whitney U test. ***p < 0.001 was regarded as statistically significant (*α* set at 0.05). In Figure 4e, ROC curves were constructed to assess the accuracy of cancer detection based on exosomal protein biomarker analysis.

## Conflict of Interest

The authors declare no conflict of interest.

## Supporting information

Supporting Information

## Data Availability

The data that support the findings of this study are available from the corresponding author upon reasonable request.
