# Supplementary material for: Portable Aptasensor Based on Parallel Rolling Circle Amplification for Tumor‐Derived Exosomes Liquid Biopsy
Source: Adv Sci (Weinh). 2024 Jun 26;11(32):2403371. doi: 10.1002/advs.202403371 (PMC11348067; doi:10.1002/advs.202403371)
Supplement: Supplementary file 1 — Supporting Information [file ADVS-11-2403371-s001.docx]

**Supporting Information**

**Portable Aptasensor Based on Parallel Rolling Circle Amplification for Tumor-Derived Exosomes Liquid Biopsy**

*Yaqin He, Xianghu Zeng, Ying Xiong, Congcong Shen, Ke Huang, and Piaopiao Chen**

Y. Q. He, X. H. Zeng, Y. Xiong, C. C. Shen, Prof. P. P. Chen*

Department of Laboratory Medicine, Med+X Center for Manufacturing, National Clinical Research Center for Geriatrics, West China Hospital, Sichuan University, Chengdu, Sichuan, 610041, China

E-mail: chenpp0828@wchscu.cn.

Prof. K. Huang

College of Chemistry and Material Science, Sichuan Normal University, Chengdu, Sichuan, 610068, China

**Content**

Title…………….……………….………………………………….………………..S-1

Content…………….……………….………………………………………………..S-2

Materials and reagents…………….……………….……………………...………...S-3

Table S1. Sequences of oligonucleotides used in the study……………………...….S-3

Figure S1. Structural formula of MB. .……………….……………………...……...S-4

Instruments...…………….………...…….…………………………………...……..S-4

Synthesis of CdTe QDs. ….……………….………………………………………...S-5

Scheme S1-S3 Handheld instrument and test strips…...……….…………………...S-5

Cell culture and exosome isolation………………………………………………….S-6

Agarose gel electrophoresis……………….…………………………………….......S-7

Steps of mucin 1 and PD-L1 analysis. …….……………………………………......S-8

Nanoparticle tracking analysis………..…….………………………………..……...S-9

Capillary western immunoassay…….……………...…………………..…………...S-9

Figure S2-S4 Optimization of mucin 1 and PD-L1 analysis conditions………..…S-10

Steps for analyzing proteins with handheld instrument……………….…………...S-12

The calculation of limit of detection……………………………………………….S-12

Table S2. Comparison of methods for the determination of mucin 1/PD-L1……...S-13

Table S3. Comparison of different methods for the determination of exosomes.....S-14

Figure S5 Analytical performance in serum matrix……………………………….S-15

Table S4. Diagnosis information and FL test results of exosomes derived from clinical patients…………….……………….…………………………………...………….S-16

References…………….……………….…………………………………...………S-17

**Materials and Reagents.**

Sodium chloride (NaCl) was purchased from Aladdin Reagent Co., Ltd (Shanghai, China). Bovine serum albumin (BSA), and agarose were bought from Sangon Biotech Co., Ltd (Shanghai, China). 4SGelred and DNA markers (50 to 500 bp) were ordered from BBI Co., Ltd (Shanghai, China). 6 × DNA loading dye was purchased from Thermo Fisher Technology (Waltham, MA, USA). Roswell Park Memorial Institute (RPMI) 1640 medium, penicillin/streptomycin and fetal bovine serum (FBS) were purchased at Gibco Invitrogen Co., Ltd (California, USA). Immunoglobulin G (IgG), lysozyme, papain, human serum albumin (HSA), pepsin, streptavidin (SA), interferon gamma (IFN-*γ*), trypsin, transferrin, human immunodeficiency virus (HIV) p24, tetanus toxoid, glucose oxidase (GOD), thrombin, nuclear matrix protein 22 (NMP-22) and glypican 3 (GPC3) ordered from Sigma-Aldrich (St. Louis, MO, USA). Anti-mucin 1, Anti-PD-L1, Anti-CD9, Anti-CD63 and Anti-CD81 antibodies were purchased from R&D systems (Minneapolis, MN, USA). Ultra-clean centrifuge tubes (25 × 89 mm, 38.5 mL, sterile) were obtained from Beckman Coulter (Indianapolis, Indiana, USA). Water used in all experiments was purified by Water Purification System (Chengdu Ultrapure Technology Co., Ltd., Chengdu, China) with a resistivity of 18.25 MΩ·cm. All reagents used in this study were of analytical grade or better and did not require further purification. All solutions were stored in a refrigerator at 4 °C before use. Clinical whole blood samples and serum were donated by West China Hospital of Sichuan University and approved by the Biomedical Ethics Committee of West China Hospital of Sichuan University (Chengdu, China, approval number: 20191045).

**Table S1.** Sequences of oligonucleotides used in the study

| name | sequence (5'-3') |
| --- | --- |
| PD-L1 aptamer | ACG GGC CAC ATC AAC TCA TTG ATA GAC AAT GCG TCC ACT GCC CGT |
| Padlock-1 | p-TGTCTATCAA TG ATAT GGGGGG TCGCAT GGGGGG TACAGAT GGGGGG TCGCAT GGGGGG CCTA AC GGG CAG TGG ACG CAT |
| Mucin 1 aptamer | GCA GTT GAT CCT TTG GAT ACC CTG GAG TGA |
| Padlock-2 | CCA AAG GAT CCTA AAAAAA TCGCAT AAAAAA TACAGCT AAAAAA TCGCAT AAAAAA CCTA TC ACT CCA GGG TAT |
| HP1 | TTTTTTTCGCATTATTACTCACGGTACGATTTTTT |
| C30 | CCC CCC CCC CCC CCC CCC CCC CCC CCC CCC |

The underlined and wavy lines represented where aptamer and padlock were complementary, and the red color represented the complementary sequences of C-C DNA and T-T DNA.


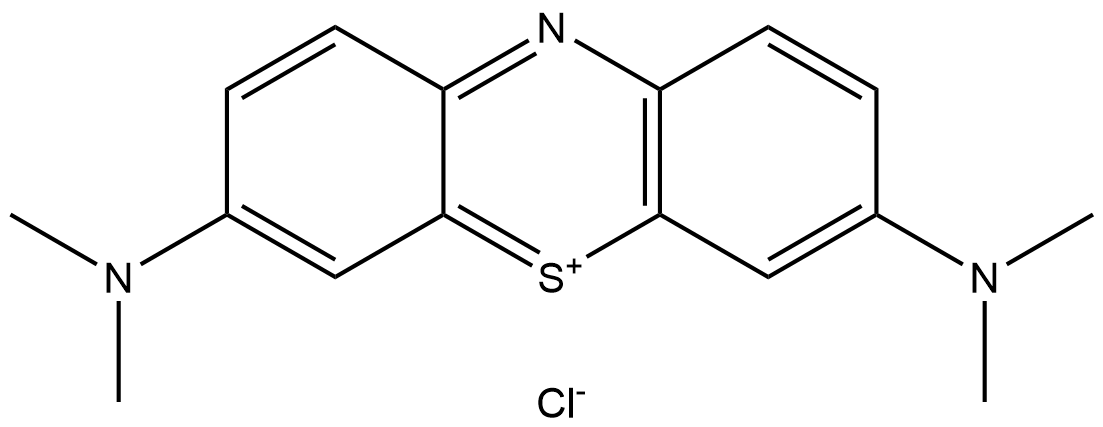


**Figure S1**. Structural formula of methylene blue (MB).

**Instruments.**

UV absorption and fluorescence spectra of CdTe quantum dots (QDs) and MB were recorded by Duetta spectrophotometer (HORIBA Canada Inc). Transmission electron microscopy (TEM) of QDs, Hg^2+^ + QDs was performed by Tecnai G2F20 STWIN TEM (FEI, USA) at the accelerating voltage of 200 kV. Atomic force microscopy (AFM) was examined on Bruker Dimension Icon AFM (Germany). Ultracentrifuge measurements were performed on an Optima XPN-100 ultracentrifuge (Beckman Coulter, Inc. Indianapolis, IN, USA) using the Type 32Ti rotor. An inkjet printer (Hewlett Packard, hp1112) was used to print templates onto chromatography paper (Whatman 3030-861, U.K.). Paper cutter (Deli, Ningbo, China) was used to cut the chromatography paper.

**Synthesis of CdTe QDs.**^[1-3]^ The CdTe QDs were synthesized referring to the previously reported method. First, a 50 mL solution contained CdCl_2_ (0.5 mmol) and trisodium citrate (0.2 g) was prepared. Then, MPA (52 μL) was instantly added into above solution, and the solution pH was adjusted to 10.5 with NaOH. Later, Na_2_TeO_3_ (0.1 mmol) and KBH_4_ (50 mg) were added into the above solution and refluxed for different time to obtain the CdTe QDs. Subsequently, high purity of CdTe QDs was obtained *via* precipitating with *n*-propanol and centrifuging (11000 rpm). The purified red and yellow CdTe QDs were redispersed in high-purity water before use.


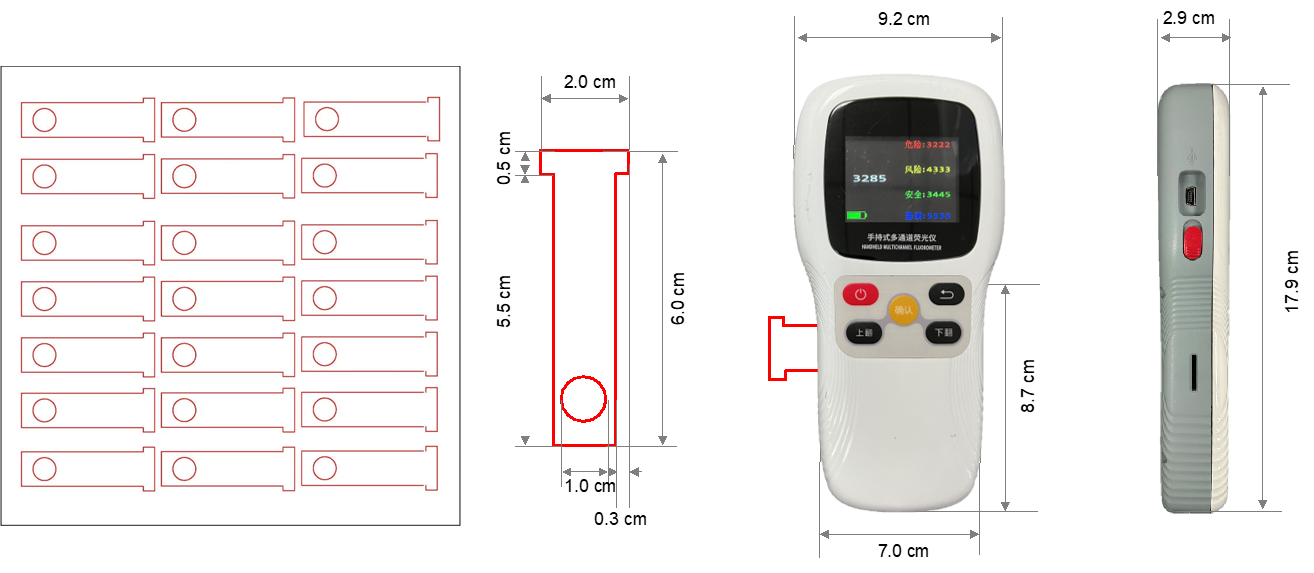


**Scheme S1.** Test strips and handheld instrument.


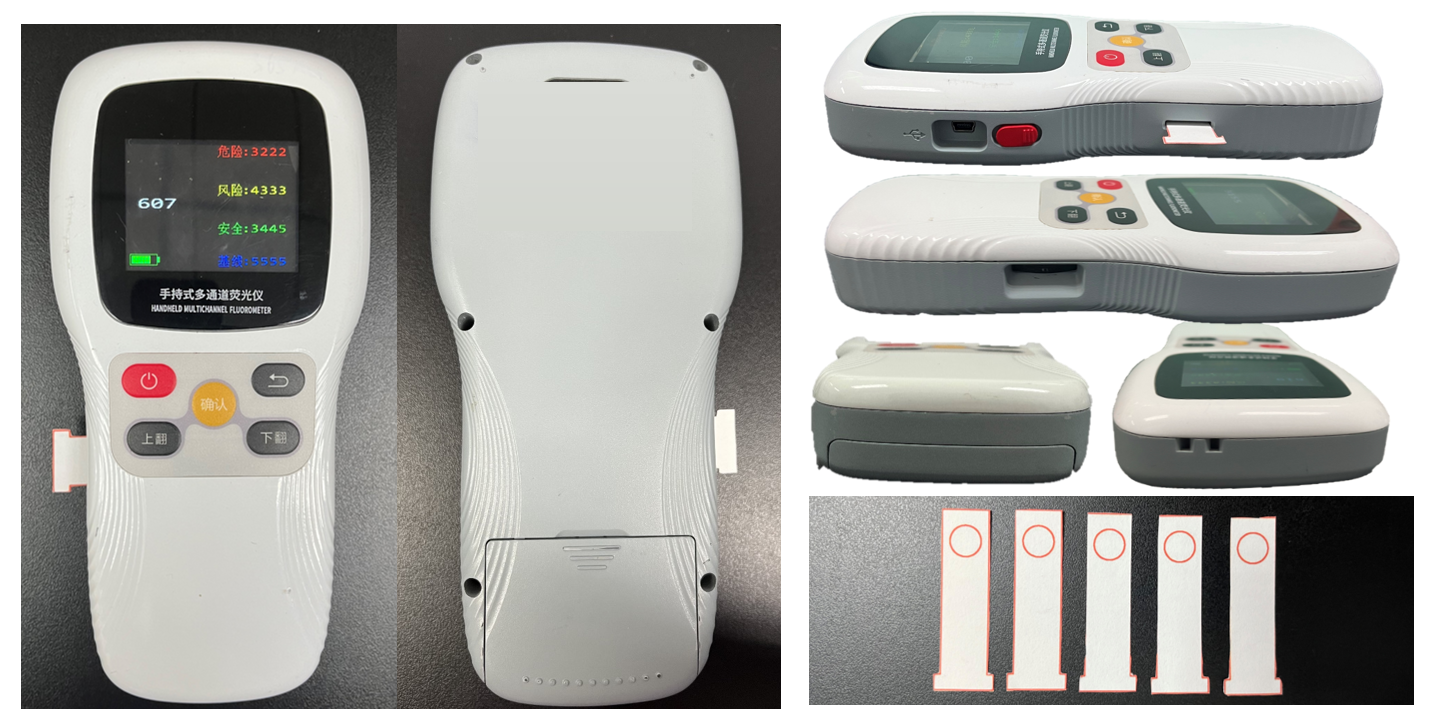


**Scheme S2.** Physical images of the handheld fluorometer and test strips.


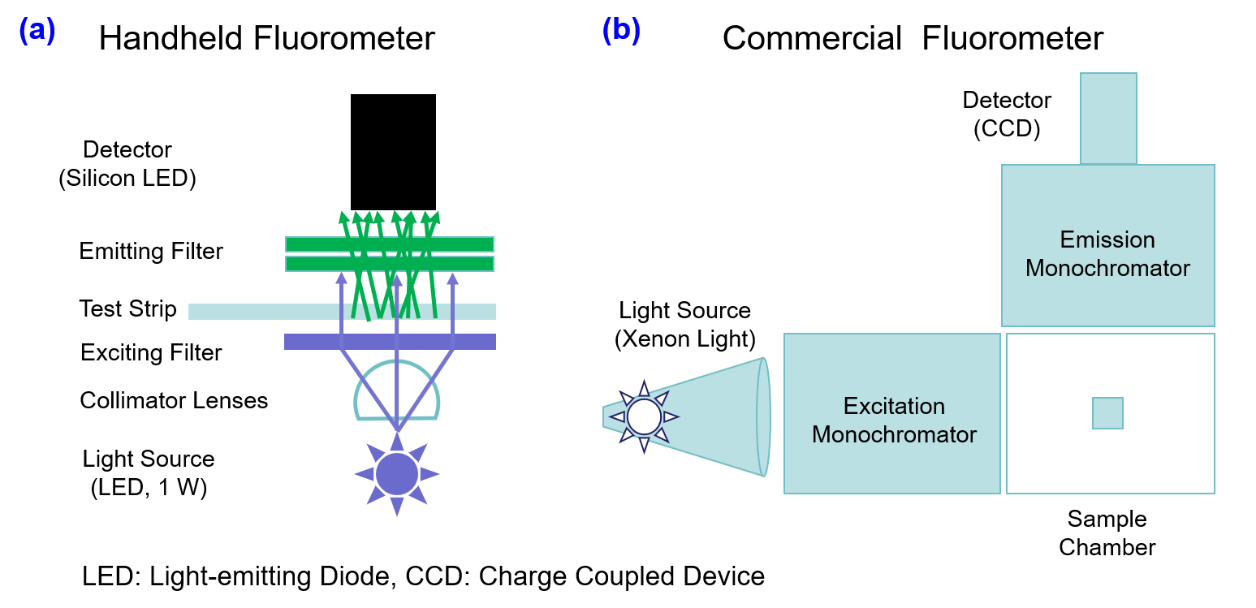


**Scheme S3.** Comparison of optical pathway diagrams of handheld fluorometers and commercial fluorometers.

**METHODS**

**Cell Culture and Exosome Isolation.** A549 cells were cultured in Roswell Park Memorial Institute (RPMI) 1640 medium containing 10% (v/v) FBS and 1% penicillin-streptomycin at 37 °C with 5% CO_2_. When the cells were in the logarithmic growth phase, they were digested into single cell suspension. The cells were then washed twice with pure RPMI 1640 medium without additives. Finally, the cells were incubated in serum-free RPMI 1640 medium for 48 hours.

Afterwards, the cell supernatant was collected in a 50 mL centrifuge tube to extract exosomes. First, the cells were centrifuged at 500 g for 10 minutes at 4 °C to precipitate cells and cellular debris. To further remove cellular debris and apoptotic bodies, the supernatant was removed and then centrifuged at 2,000 g for 20 minutes at 4 °C. The supernatant was then centrifuged at 10,000 g for 30 minutes at 4 °C to remove large extracellular vesicles. The supernatant was then filtered through a 0.22 µm membrane to minimize particulate contamination. After filtration, the supernatant was rinsed with PBS and centrifuged at 150,000 g for 2 hours at 4 °C, and this process was repeated twice. Finally, the isolated exosomes were resuspended in 200 μL of PBS and stored at -80 °C. The exosomes were characterized using TEM, NTA and western blotting.

**Agarose Gel Electrophoresis**

The instrument and assay of agarose gel electrophoresis (the agarose concentration was 3%) were purchased from Sangon Biotech Co., Ltd. (Shanghai, China).

2.4 g of agarose was weighed and dissolved in 80 mL of 1x TAE buffer, then 8 μL of 2000x 4SGelred was added. After electrophoresis, it was visualized with a gel imager (Gel Doc^TM^ EZ Imager, Bio-Rad Laboratories, USA).

For PD-L1:

From left to right: (1) marker (500-50 bp); (2) PD-L1 aptamer; (3) Padlock-1; (4) Aptamer + padlock-1 (dsDNA-1); (5) dsDNA-1 + T4 ligase; (6-9) dsDNA-1 + T4 ligase + phi 29 polymerase + PD-L1 (0, 10 ag/mL, 1 fg/mL, 100 fg/mL).

For mucin 1:

From left to right: (1) marker (500-50 bp); (2) Mucin 1 aptamer; (3) Padlock-2; (4) Aptamer + padlock-2 (dsDNA-2); (5) dsDNA-2 + T4 ligase; (6-9) dsDNA-2 + T4 ligase + phi 29 polymerase + mucin 1 (0, 10 ag/mL, 1 fg/mL, 100 fg/mL).

**Steps of mucin 1 and PD-L1 analysis.**

The reaction was performed by adding 10 μM 4 μL Padlock-1, 10 μM 4 μL PD-L1-aptamer and 800 mM 2 μL NaCl to 10 μL of water, mixing well. Then placing in the polymerase chain reaction (PCR) instrument and setting the annealing program: 95 ℃ for 5 min; 65 ℃ for 2 min; 60 ℃ for 6 min; 60 to 20 ℃, -0.5 °C/30 s; 4 ℃ for 10 min. After that, 40 μL different concentrations of PD-L1 were added reacted for 30 min. For mucin 1, the procedure was the same except that it was changed to Padlock-2 and mucin 1-aptamer. Add 40 μL of mucin 1 and react for 30 min.

Then, mix the two reaction solutions, add 10 μL 10 × T4 buffer and 5 U/μL 2 μL T4 DNA ligase, and react at 37 °C for 1 hour. After incubation put into 65 ℃ for 10 min to inactivate T4 ligase. Add 10 μL 10 × phi29 buffer, 10 U/μL 2 μL phi29 DNA polymerase, 800 mM 10 μL NaCl, 25 mM 4 μL dNTP and 20 mg/mL 1 μL BSA and incubate at 37 ℃ for 90 min. After incubation, put into 65 ℃ for 10 min. Add 50 μM 5 μL Hg^2+^ and react for 1 h to form a T- Hg^2+^-T structure. Last, add 50 μM 4 μL MB and 2 μL QDs stock solution and react for 5 min and measure fluorescence.

**Nanoparticle Tracking Analysis (NTA).**

Particle concentration and size distribution of exosomes isolated from cells and plasma samples were analyzed by ZetaView (Particle Metrix, Meerbusch, Germany). Each sample was first diluted 100-fold with PBS. Instrument parameters were: temperature 24 °C, shutter 70, sensitivity 70, Min Brightness 20, Max Area and Min Area are 1000 and 5 respectively. Data acquisition and analysis of the nanoparticles was performed by the Nanoparticle Tracking Analysis Software (ZNTA) v 8.05.04, and each reported value was averaged over three replicates.

**Capillary Western Immunoassay.**

RIPA buffer (Thermo Fisher Scientific, 89900) was used to extract exosomal lysates with phenylmethylsulfonyl fluoroproteinase inhibitor. BCA Protein Assay Kit (Thermo Fisher Scientific, 23227) was used to detect protein concentration. For capillary-based immunoassays, load exosome lysates into Jess/Wes 12-230 kDa pre-filled plates (ProteinSimple, SM-W002). Specific proteins are detected using commercially available antibodies against anti-CD9, anti-CD63, anti-CD81, anti-mucin 1, and anti-PD-L1. Conjugated antibodies are detected and visualized using the anti-rabbit or anti-mouse HRP detection modules (ProteinSimple, MS-001) and the data is analyzed by Compass for Simple Western software (ProteinSimple).

**Optimization of Mucin 1 and PD-L1 Analysis Conditions.**

In order to improve the analytical performance, the experimental conditions of rolling circle amplification (RCA) were first optimized by measuring mucin 1 and PD-L1 simultaneously. As shown in Figure S2a and S2b, 2 μL of T4 ligase (5 U/L) was added and incubated for 60 minutes sufficient to form circular template. The appropriate amount of phi29 DNA polymerase, volume of dNTPs and incubation time contribute to the efficient completion of RCA. Experimentally, the optimal conditions were 2 μL phi29 DNA polymerase (Figure S2c, 10 U/μL), 4 μL dNTPs (Figure S2d, 25 mM) and 90 minutes of amplification time (Figure S2e).


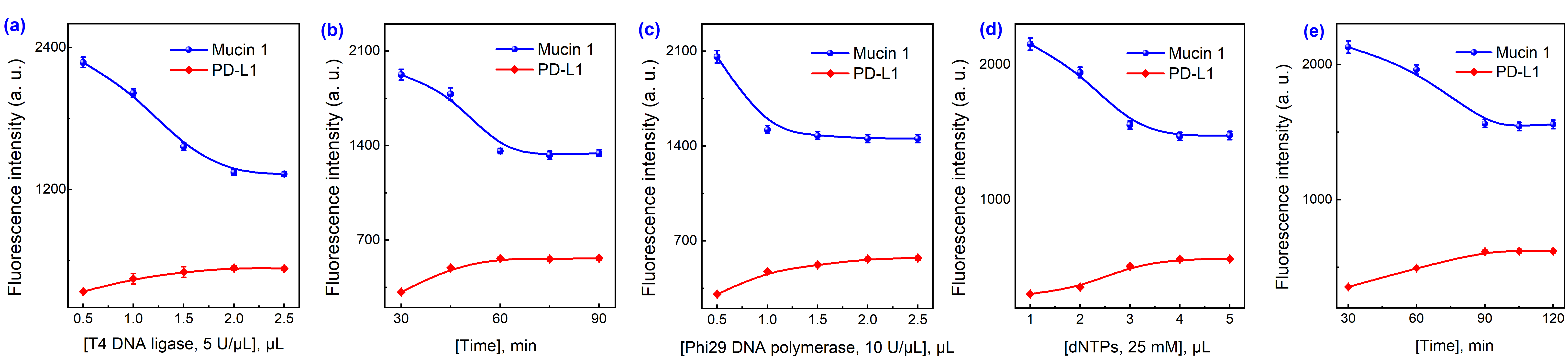


**Figure S2.** Optimization of RCA experimental conditions. (a) The volume of T4 ligase. (b) Time of circular template formation. (c) The volume of phi29 DNA polymerase. (d) The volume of dNTPs. (e) Time of RCA. Error bars were estimated from three replicate measurements.

After RCA was completed, mucin 1 was detected by the difference in fluorescence obtained from the reaction of QDs with Hg^2+^ and T-Hg^2+^-T complex. The maximum fluorescence signal difference between 1 fg/mL mucin 1 and blank groups was achieved at Hg^2+^ concentration of 50 μM (Figure S3a-b, 5 μL). The formation time of T-Hg^2+^-T was 1 hour (Figure S3c). As shown in Figure S3d and S3e, the optimal amount of QDs was 2 μL, at which point the signal difference was greatest (1 fg/mL mucin 1 *vs.* blank). In addition, 5 min was sufficient for the QDs to fully react with Hg^2+^ (Figure S3f).


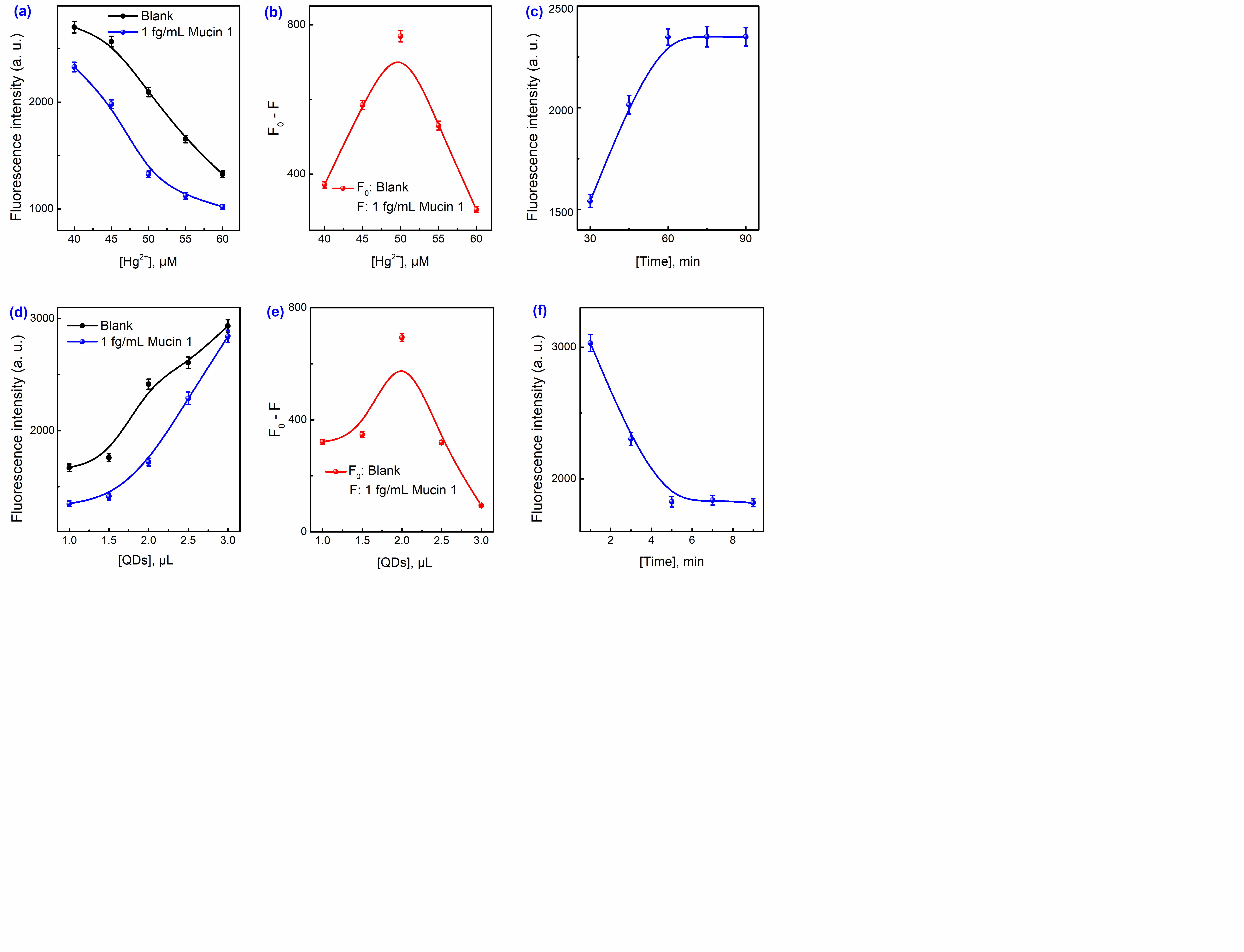


**Figure S3.** Optimization of mucin 1 analysis conditions. (a, b) Concentration of Hg^2+^. (c) Time of T-Hg^2+^-T structure formation. (d, e) Amount of QDs. (f) Reaction time of QDs and Hg^2+^. Error bars were estimated from three replicate measurements.

At the same time, PD-L1 was detected by the reaction of the C base generated by RCA with MB. The results showed that a significant difference in peak fluorescence signal between 1 fg/mL mucin 1 and the blank group could be observed by adding 50 μM MB for 5 minutes of reaction (Figure S4a-c).


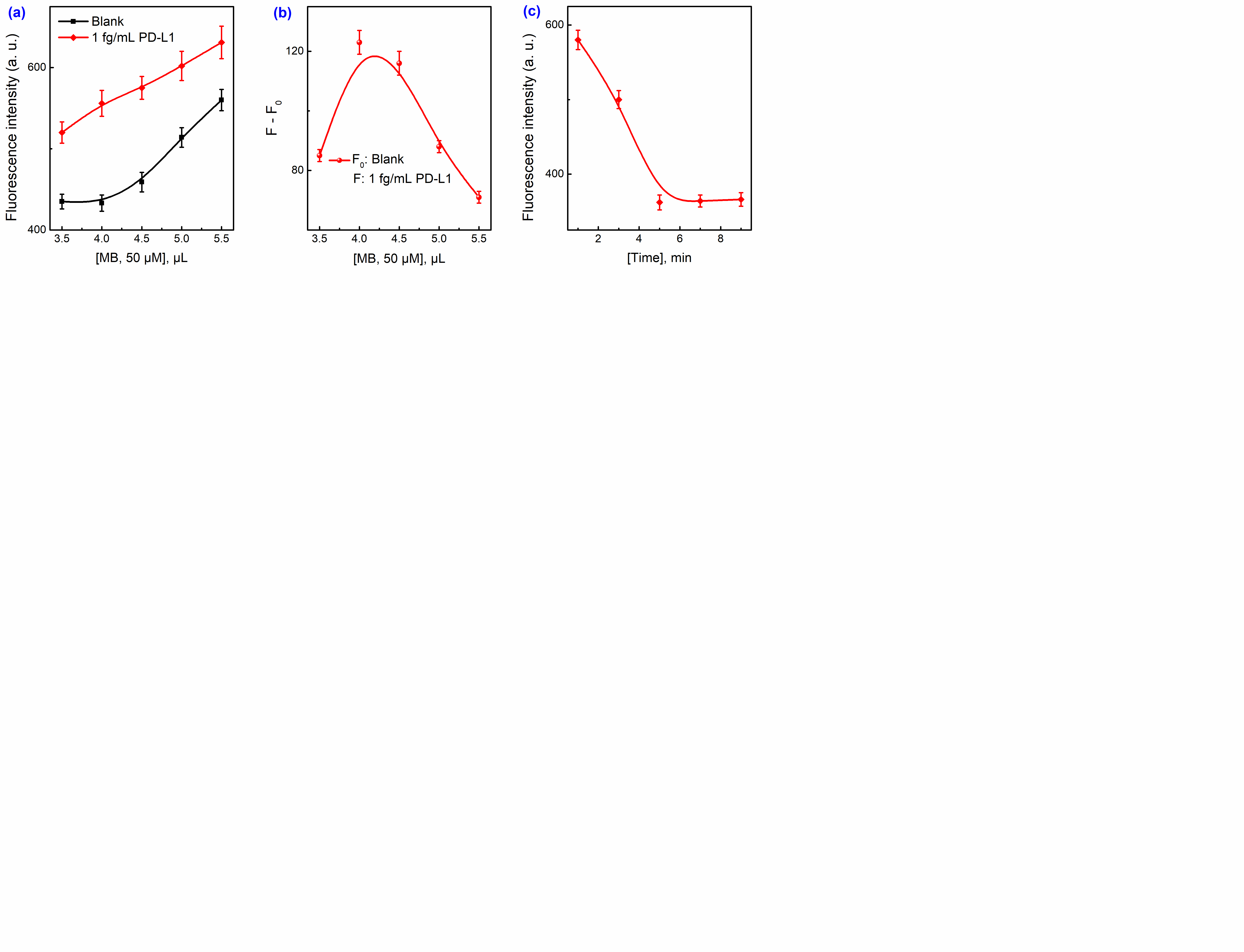


**Figure S4.** Optimization of PD-L1 analysis conditions. (a, b) Concentration of MB. (c) Reaction time of MB. Error bars were estimated from three replicate measurements.

**Steps for analyzing proteins with handheld instrument.**

Considering the loading capacity for fluorescent materials and storage conditions of the test strips, we chose Whatman chromatography paper (20 × 20 cm) for testing. First, print on chromatography paper according to the template (Scheme S1). Then cut into test strips using a paper cutter and store sealed at room temperature. After adding 50 μM 4 μL MB and 2 μL QDs stock solution to the reaction system for 5 min, 15 μL of reaction solution was taken and dropped into the circular area of the test strip and immediately detected at the corresponding emission wavelength.

**The calculation of limit of detection (LOD)**

According to the literature,^[4-6]^ the LOD was calculated by giving a fluorescence signal three times standard deviation (SD) lower than the background signal in the absence of a target.

First, the fluorescence signal-target concentration curve obtained by least-square root linear regression was the standard curve. The standard curve of was plotted as:

***Y* = 𝐴 Log*C* + *B* (1)**

Where A and B were the slope and intercept of the standard curve, respectively. *C* was the concentration of target, and Y represented the corresponding fluorescence value.

When:

**𝑌 = 𝑌_blank_ - 3𝑆𝐷 (2)**

Where SD was the standard deviation and *Y*_blank_ was the fluorescence signal of the blank sample (without target).

The LOD was calculated as:

***LOD* = 10 ^[( 𝑌^_blank_ ^- 3^*^SD^* ^) –^ *^B^*^] /^ *^A^* (3)**

**Table S2.** Comparison of methods for the determination of mucin 1/PD-L1

| Method | System | Target | | Linear range; LOD | Homogeneity | Ref. |
| --- | --- | --- | --- | --- | --- | --- |
| ddPCR^a^ | TRACER^b^, aptamer | | PD-L1, EpCAM | 0-217.5 pg/mL;  0.0735 pg/mL | No | ^[7]^ |
| Fluorescence | Aptamer, thermophoresis | | PD-L1 | 0-353.8 pg/mL;  17.6 pg/mL | Yes | ^[8]^ |
| PEC^c^ | PEDOT/BiOBr_0.8_I_0.2_, MWCNTs/SnS_2_ | | PD-L1 | 1 pg/mL-100 ng/mL; 0.29 pg/mL | No | ^[9]^ |
| PEC | Dual heterojunction, ZnCdS/NiS/ZnCdS | | PD-L1 | 10 fg/mL-10 ng/mL;  4.02 fg/mL | No | ^[10]^ |
| SPR^d^ | MNOM@AgNCs-Apt PD-L1, antibody | | PD-L1 | 10-300 ng/mL;  3.29 ng/mL | No | ^[11]^ |
| LFAs^e^ | DNA-based scaffold | | Mucin 1 | Not mentioned; 0.3 nM | Yes | ^[12]^ |
| AIECL^f^ | TPE-UiO-66, AuNPs, DOX | | Mucin 1 | 10 fg/mL-1 ng/mL;  7.6 fg/mL | No | ^[13]^ |
| FL^g^ | CHA^h^, C-Ag^+^-C, QDs | | Mucin 1 | 1 fg/mL-1 pg/mL;  0.15 fg/mL | Yes | ^[3]^ |
| ICP-MS^i^ | CHA, nanoparticles | | Mucin 1 | 1 ag/mL-100 fg/mL;  0.3 ag/mL | Yes | ^[14]^ |
| ECL^j^ | Aptamer, Exo III,  Eu-PCP | | Mucin 1 | 1 fg/mL-10 ng/mL;  0.32 fg/mL | No | ^[15]^ |
| EC^k^ | Aptamer, Au-AAO ion channel | | Mucin 1 | 1-10^4^ fg/mL;  0.0364 fg/mL | No | ^[16]^ |
| FL | Aptamer; RCA; T-Hg^2+^-T; QDs; MB | | PD-L1, mucin 1 | 1-100 ag/mL, 0.3 ag/mL; 10-1000 ag/mL, 4 ag/mL | Yes | This work |

^a^ Droplet digital PCR; ^b^ Dual-target-specific aptamer recognition activated in situ connection system on exosome membrane combined with ddPCR; ^c^ Photoelectrochemical; ^d^ Surface plasmon resonance; ^e^ Lateral flow assays; ^f^ Aggregation-induced electrochemiluminescence; ^g^ Fluorescence; ^h^ Catalytic hairpin assembly; ^i^ Inductively coupled plasma mass spectrometry; ^j^ Electrochemiluminescence; ^k^ Electrochemistry.

**Table S3.** Comparison of different methods for the determination of exosomes

| Method | System | Identification target | Linear range; LOD (particles/mL) | Homogeneity | Ref. |
| --- | --- | --- | --- | --- | --- |
| Microfluidic | MINDS^a^ chip | CD24, EpCAM, FRα | 10^4^-10^9^; 10^4^ | No | ^[17]^ |
| Single-wavelength imaging | Optofluidic chip | CD9 | 1.23 × 10^8^-5× 10^10^;  1.23 × 10^8^ | No | ^[18]^ |
| EC^b^ | Anti CD44@IMBs; CHA^c^; cell membrane | PD-L1 mRNA | 10^3^-10^9^; 557 | No | ^[19]^ |
| EC | HiMEX^d^ | EGFR, EpCAM, CD24, etc | 10^4^-10^9^; 10^4^ | No | ^[20]^ |
| EC | Aptamer; RCA^f^; hemin/G-Quadruplex | CD63 | 4.8 × 10^3^-4.8 × 10^6^；  9.54 × 10^2^ | No | ^[21]^ |
| SERS^g^ | iREX^h^ biosensor | HER2, mucin 1, CEA | 10^7^-10^12^; 1.0-3.0 × 10^7^ | No | ^[22]^ |
| FL | Apt/C_3_N_4_ NSs | mucin 1 | 8.53 × 10^3^ - 3.01 × 10^6^; 2.5 × 10^3^ | Yes | ^[23]^ |
| FL | Branched  DNA nanostructures | PD-L1 | 10^3^-10^9^; 170 | Yes | ^[24]^ |
| LSPR^i^ | Nanoplasmonic Sandwich Immunoassay | PD-L1 | 0-400 × 10^6^; 1.2 × 10^6^ | No | ^[25]^ |
| FL (POCT) | QDs; MB; T-Hg^2+^-T; aptamer; RCA | PD-L1, mucin 1 | 10^2^-10^4^, 30; 10^3^-10^5^, 200 | Yes | This work |

^a^ multiscale integration by designed self-assembly; ^b^ electrochemical; ^c^ catalytic hairpin assembly; ^d^ high-throughput integrated magneto-electrochemical extracellular vesicle; ^f^ rolling circle amplification; ^g^ surface-enhanced Raman spectroscopy; ^h^ integrated Raman spectroscopic exosomes; ^i^ localized surface plasmon resonance.


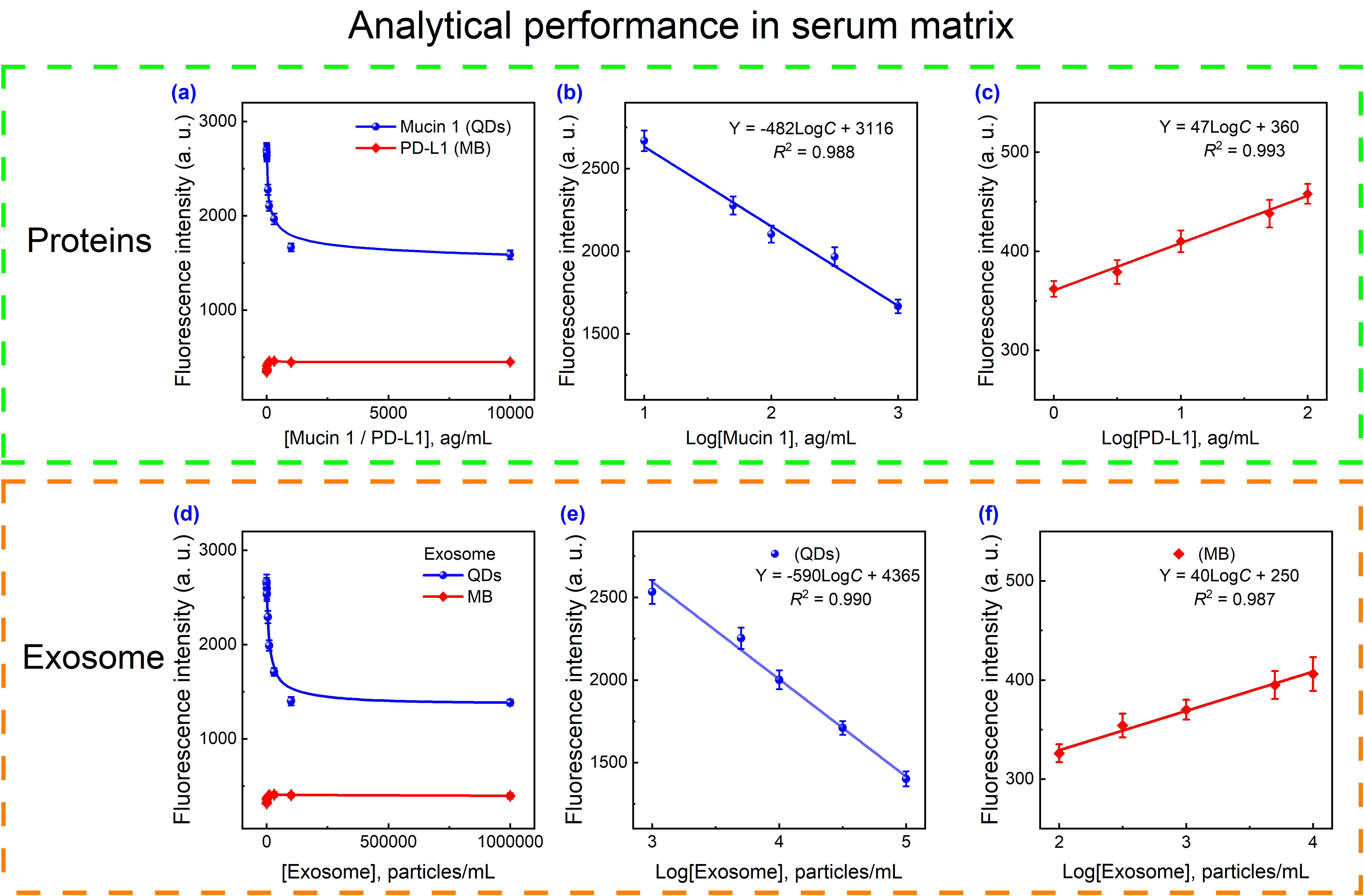


**Figure S5.** Analytical performance in serum matrix. Fluorescence values (a) for different concentrations of proteins. Linear fits for different concentrations of mucin 1 (b) and PD-L1 (c). Fluorescence values (d) for different concentrations of exosome. Linear fits of QDs (e) and MB signals (f). Error bars were estimated from three repeated measurements.

**Table S4.** Diagnosis information and fluorescence (FL) test results of exosomes derived from clinical patients (the results were the fluorescence value of mucin 1 detected *via* QDs)

| No. | Age | Sex | Clinical diagnosis | FL^a^ results | Portable instrument | Consistency |
| --- | --- | --- | --- | --- | --- | --- |
| 1 | 56 | female | positive | 2587 | 2165 | yes |
| 2 | 65 | male | positive | 2230 | 1970 | yes |
| 3 | 57 | male | positive | 2164 | 2084 | yes |
| 4 | 50 | female | positive | 1999 | 1916 | yes |
| 5 | 60 | female | positive | 1970 | 1887 | yes |
| 6 | 49 | male | positive | 2334 | 1955 | yes |
| 7 | 54 | female | positive | 2047 | 1943 | yes |
| 8 | 55 | female | positive | 2078 | 1860 | yes |
| 9 | 70 | male | positive | 2566 | 2098 | yes |
| 10 | 27 | male | positive | 2926 | 2206 | no |
| 11 | 34 | male | positive | 2001 | 2072 | yes |
| 12 | 40 | female | positive | 2598 | NT^b^ | yes |
| 13 | 29 | female | positive | 2351 | NT | yes |
| 14 | 61 | male | positive | 2350 | NT | yes |
| 15 | 31 | male | positive | 2187 | NT | yes |
| 16 | 62 | male | positive | 2276 | NT | yes |
| 17 | 35 | male | positive | 1936 | NT | yes |
| 18 | 50 | female | positive | 2079 | NT | yes |
| 19 | 34 | male | positive | 1678 | NT | yes |
| 20 | 38 | female | positive | 1808 | NT | yes |
| 21 | 52 | male | positive | 2048 | NT | yes |
| 22 | 39 | female | positive | 2195 | NT | yes |
| 23 | 56 | female | positive | 2669 | NT | no |
| 24 | 40 | female | positive | 2291 | NT | yes |
| 25 | 55 | male | positive | 2467 | NT | yes |
| 26 | 29 | male | negative | 3041 | 2487 | yes |
| 27 | 63 | female | negative | 2768 | 2435 | yes |
| 28 | 61 | female | negative | 2851 | 2320 | yes |
| 29 | 42 | female | negative | 2881 | 2367 | yes |
| 30 | 36 | male | negative | 2937 | 2298 | yes |
| 31 | 39 | male | negative | 2448 | NT | no |
| 32 | 46 | female | negative | 2775 | NT | yes |
| 33 | 58 | male | negative | 3112 | NT | yes |
| 34 | 34 | male | negative | 2769 | NT | yes |
| 35 | 61 | male | negative | 3007 | NT | yes |
| 36 | 44 | female | negative | 2874 | NT | yes |
| 37 | 57 | male | negative | 2688 | NT | yes |
| 38 | 52 | female | negative | 2919 | NT | yes |
| 39 | 39 | male | negative | 2669 | NT | yes |
| 40 | 43 | male | negative | 2774 | NT | yes |

^a^ Fluorescence; ^b^ Not tested.

**References**

[1] X. Jiang, Y. J. Bai, Q. L. Liu, L. Yan, T. T. Long, M. Li, J. Huang, B. W. Ying and P. P. Chen, *Anal. Chim. Acta* **2023**, *1237*, 340586.

[2] P. P. Chen, Y. M. Meng, T. Y. H. Liu, W. Peng, Y. Gao, Y. Q. He, R. L. Qu, C. Y. Zhang, W. Hu and B. W. Ying, *ACS Nano* **2023**, *17*, 6998.

[3] P. P. Chen, Y. Wang, Y. Q. He, K. Huang, X. Wang, R. H. Zhou, T. Y. H. Liu, R. L. Qu, J. Zhou, W. Peng, M. Li, Y. J. Bai, J. Chen, J. Huang, J. Geng, Y. Xie, W. Hu and B. W. Ying, *ACS Nano* **2021**, *15*, 11634.

[4] J. Y. Luan, A. Seth, R. Gupta, Z. Y. Wang, P. Rathi, S. S. Cao, H. Gholami Derami, R. Tang, B. G. Xu, S. Achilefu, J. J. Morrissey and S. Singamaneni, *Nat. Biomed. Eng.* **2020**, *4*, 518.

[5] W. Ma, H. H. Yin, L. G. Xu, X. L. Wu, H. Kuang, L. B. Wang and C. L. Xu, *Chem. Commun.* **2014**, *50*, 9737.

[6] Y. Q. He, Z. X. Zhan, L. Yan, C. Y. Wu, Y. Wang, C. C. Shen, K. Huang, Z. L. Wei, F. Lin, B. W. Ying, W. M. Li and P. P. Chen, *ACS Nano* **2024**, *18*, 5017.

[7] B. Q. Lin, T. Tian, Y. Z. Lu, D. Liu, M. J. Huang, L. Zhu, Z. Zhu, Y. L. Song and C. Y. Yang, *Angew. Chem. Int. Ed.* **2021**, *60*, 7582.

[8] M. J. Huang, J. J. Yang, T. Wang, J. Song, J. L. Xia, L. L. Wu, W. Wang, Q. Y. Wu, Z. Zhu, Y. L. Song and C. Y. Yang, *Angew. Chem. Int. Ed.* **2020**, *59*, 4800.

[9] T. T. Wang, Y. Q. Ran, Y. F. He, L. Shi, B. Z. Zeng and F. Q. Zhao, *Biosens. Bioelectron.* **2023**, *237*, 115558.

[10] Y. M. Li, Z. S. Li, H. B. Tan, Y. Y. Li, F. Tang, P. Wang, Y. Y. Li, H. Liu, P. Q. Zhao and Q. Liu, *Sens. Actuators, B* **2023**, *376*, 133044.

[11] X. Huang, Z.-H. Zhang, J. Chen, Z. H. Mao, H. Zhu, Y. W. Liu, Z. Z. Zhu and H. X. Chen, *Biosens. Bioelectron.* **2021**, *189*, 113385.

[12] S. Brannetti, S. Gentile, A. Chamorro-Garcia, L. Barbero, E. Del Grosso and F. Ricci, *Angew. Chem. Int. Ed.* **2023**, *62*, e202313243.

[13] X. Y. Xiong, C. Y. Xiong, Y. Gao, Y. Xiao, M.-M. Chen, W. Wen, X. H. Zhang and S. F. Wang, *Anal. Chem.* **2022**, *94*, 7861.

[14] Y. Wang, X. Chen, X. Shen, Y. Q. He, Z. X. Zhan, C. X. Liu, Y. Xie, F. Lin, K. Huang and P. P. Chen, *Anal. Chem.* **2023**, *95*, 14244.

[15] Q. Han, C. Wang, P. K. Liu, G. Zhang, L. Song and Y. Z. Fu, *Biosens. Bioelectron.* **2021**, *191*, 113422.

[16] M. Y. Pan, J. R. Cai, S. Li, L. G. Xu, W. Ma, C. L. Xu and H. Kuang, *Anal. Chem.* **2021**, *93*, 4825.

[17] P. Zhang, X. Zhou, M. He, Y. Q. Shang, A. L. Tetlow, A. K. Godwin and Y. Zeng, *Nat. Biomed. Eng.* **2019**, *3*, 438.

[18] Y. Jahani, E. R. Arvelo, F. Yesilkoy, K. Koshelev, C. Cianciaruso, M. De Palma, Y. Kivshar and H. Altug, *Nat. Commun.* **2021**, *12*, 3246.

[19] Y. Cao, X. M. Yu, T. Y. Zeng, Z. Y. Fu, Y. Y. Zhao, B. B. Nie, J. Zhao, Y. M. Yin and G. X. Li, *J. Am. Chem. Soc.* **2022**, *144*, 13475.

[20] J. Park, J. S. Park, C.-H. Huang, A. Jo, K. Cook, R. Wang, H.-Y. Lin, J. Van Deun, H. Y. Li, J. Min, L. Wang, G. Yoon, B. S. Carter, L. Balaj, G.-S. Choi, C. M. Castro, R. Weissleder and H. Lee, *Nat. Biomed. Eng.* **2021**, *5*, 678.

[21] R. R. Huang, L. He, Y. Y. Xia, H. P. Xu, C. Liu, H. Xie, S. Wang, L. J. Peng, Y. F. Liu, Y. Liu, N. Y. He and Z. Y. Li, *Small* **2019**, *15*, 1900735.

[22] X. M. Su, X. Y. Liu, Y. C. Z. Xie, M. Y. Chen, C. Zheng, H. Zhong and M. Li, *ACS Nano* **2023**, *17*, 4077.

[23] M.-X. Liu, H. Zhang, X.-W. Zhang, S. Chen, Y.-L. Yu and J.-H. Wang, *Anal. Chem.* **2021**, *93*, 9002.

[24] X. Y. Li, X. M. Li, X. X. Cheng, X. T. Bian, B. Shen, X. J. Ding and S. J. Ding, *ACS Sens.* **2022**, *7*, 3571.

[25] C. Y. Wang, C.-H. Huang, Z. Q. Gao, J. L. Shen, J. C. He, A. MacLachlan, C. Ma, Y. Chang, W. Yang, Y. X. Cai, Y. Lou, S. Y. Dai, W. Q. Chen, F. Li and P. Y. Chen, *ACS Sens.* **2021**, *6*, 3308.
